# Localised structuring of metal-semiconductor cores in silica clad fibres using laser-driven thermal gradients

**DOI:** 10.1038/s41467-022-29975-1

**Published:** 2022-05-13

**Authors:** Seunghan Song, Fredrik Laurell, Bailey Meehan, Thomas W. Hawkins, John Ballato, Ursula J. Gibson

**Affiliations:** 1 PoreLab and Physics Department, Norwegian University of Science and Technology, 7491 Trondheim, Norway; 2 KTH Applied Physics, 10691 Stockholm, Sweden; 3 Department of Materials Science and Engineering, Clemson University, Clemson, SC 29634 USA

**Keywords:** Materials for optics, Materials for devices, Fibre optics and optical communications

## Abstract

The molten core drawing method allows scalable fabrication of novel core fibres with kilometre lengths. With metal and semiconducting components combined in a glass-clad fibre, CO_2_ laser irradiation was used to write localised structures in the core materials. Thermal gradients in axial and transverse directions allowed the controlled introduction, segregation and chemical reaction of metal components within an initially pure silicon core, and restructuring of heterogeneous material. Gold and tin longitudinal electrode fabrication, segregation of GaSb and Si into parallel layers, and Al doping of a GaSb core were demonstrated. Gold was introduced into Si fibres to purify the core or weld an exposed fibre core to a Si wafer. Ga and Sb introduced from opposite ends of a silicon fibre reacted to form III-V GaSb within the Group IV Si host, as confirmed by structural and chemical analysis and room temperature photoluminescence.

## Abstract (web summary)

The authors show that micron size fibers comprised of semiconductor and metallic materials can be purified or doped, recrystallized or have the constituents rearranged into a variety of structures with device prospects.

## Introduction

Novel core fibres as a platform for electronic and optical applications are of growing interest, as evidenced by a number of recent review articles^1–7^, and structuring of mixed material microstructures is an ongoing interest for the solar^8–10^, electronics^11–14^ and photonic^15,16^ communities. There are two temperature ranges that are encompassed in the fibre research, one with polymer-clad multimaterials, and one with conventional semiconductors in glass. The lower temperature systems are able, for example, to incorporate previously fabricated diodes into the preform, connected by wire that is spooled into the fibre as it draws^17^, while purely inorganic systems such as silicon-in-silica fibres perform a variety of optical functions, can be used at higher temperatures, and are chemically robust. Post-processing of as-drawn fibre has been employed to improve the properties of core materials, in particular for Group IV semiconductors^18^, which are typically inhomogeneous and/or polycrystalline as drawn, with impurities at the grain boundaries^19^. Dramatic improvement in the optical transparency^20,21^ of silicon core fibres has been made, and non-linear optical behaviour is now well established^22^.

One of the driving forces for the development of semiconductor cores has been the potential for optoelectronic devices, and prototype devices have been made in Group IV semiconductor core fibres^23,24^, using Si as well as SiGe. Detector development and solar conversion are of particular interest, but material quality, doping and electrode formation are challenging^8,23,25,26^ due to the high processing temperatures involved; for example, glass distortion during Plateau-Rayleigh capillary instability breakup of the core has been used to contact in-fibre electrodes^24^. Fabrication of local devices is an ongoing challenge.

Much research has focused on using laser heating to recrystallise and homogenise the core^27^, in some cases with multiple beams to create a symmetric thermal profile^28,29^, but nonuniform heating is also of interest. Using unidirectional laser illumination with airflow cooling, radial structuring of the core composition profile has recently been demonstrated in the silicon-germanium alloy system^30,31^, and transverse thermal gradients have been used to transport semiconductor microparticles within the glass cladding of a fibre^32^.

Post-draw spatial segregation has also been demonstrated for composite cores such as the (pseudo)eutectic systems Si-GaSb^33^ and Au-Si^21^. During processing, laser-induced thermal gradients create a higher concentration of the lower melting point material at the laser focus through the thermomigration process. This liquid alloy can then be translated along the core of the fibre in a miniature version of travelling solvent float zone (TSFZ) recrystallisation or can be solidified in a controlled manner to add structure to the core. As the laser power is reduced, the lower melting point constituent segregates from the silicon, and geometrical arrangement of the two components within the core is possible.

**Fig. 1:**
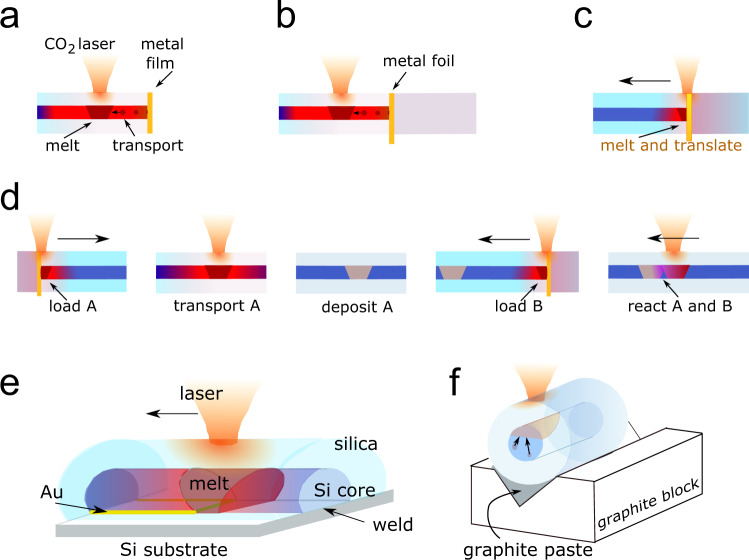
Schematic of sample preparation methods. **a** Thermomigration of metal from a film deposited on the fibre end, driven by CO_2_ laser heating, **b** thermomigration from a metal foil pressed against the semiconductor core fibre. These methods allow incorporation of small amounts of metal for zone refining or doping. **c** Bulk dissolution of a foil into the semiconductor core, followed by translation, useful where a longitudinal electrode formation is desired, **d** sequential introduction steps used to drive in-core formation of compounds, **e** use of solvent (Au) film and travelling melt zone to weld the silicon core of a fibre to a silicon substrate, and (**f**) use of graphite as a thermal sink to establish a strong vertical component to the thermal gradient, allowing thermomigration segregation of eutectic components within the fibre core.

Thermomigration in semiconductors occurs when the composition and temperature of an alloy are nonuniform, with a solvent phase, typically a low melting point metal alloy, forming a droplet that will migrate through the solid host material towards the highest temperature. The solubility of the majority constituent (Si, in most studies) is higher at the hot side of the molten region, and the resulting concentration gradient drives diffusion across the droplet. Supersaturation at the cooler side of the melt leads to solidification, and this mass displacement translates the melt zone towards the high temperature. The process can be used to rearrange the components of a multimaterial fibre. Figure 1 schematically presents several device-oriented experiments that were performed using laser-generated thermal gradients; in panels a–c, introduction of one or more metallic elements into silicon core fibres is followed by translation of the melt zone within the fibre, d illustrates reactive formation of compounds within the silicon host, e demonstrates welding of semiconductor core fibres to a substrate using a thin solvent layer to seed local melting and attachment of the fibre core, and f demonstrates the use of transverse thermal gradients to segregate immiscible components, thus either establishing either a conductive electrode along the side of the fibre core or a longitudinal structure with two semiconductors side by side.

In early studies, thermomigration was explored as a potential method for localised doping^34,35^, device formation^36^, recrystallisation, and joining of silicon wafers^37^. Thermal gradients were typically less than 100 K cm^−1^, and large cross-section samples led to poorly controlled trajectories^38^. A useful overview of the process is provided by Cline and Anthony^39^ based on their doping of semiconductors, primarily Si. Studies of thermomigration continue, as the temperature gradients across microelectronic bump solders^40^ can exceed 1000 K cm^−1^, and associated segregation can lead to failure. The process is also employed for growth and modification of nanowires^41–43^.

In a simplified model, the migration rate, *v*, can be written as:

1 $${v}_{{{{{{\rm{droplet}}}}}}} 	=\frac{1}{{C}_{{{{{{{\rm{Si}}}}}}}}^{s}}\left({-D}_{{{{{{\rm{{Si}}}}}}}}\nabla {C}_{{{{{{\rm{{Si}}}}}}}}^{l}+\frac{{D}_{{{{{{\rm{{Au}}}}}}}}{C}_{{{{{{\rm{{Si}}}}}}}}^{l}}{{C}_{{{{{{\rm{{Au}}}}}}}}^{l}}\nabla {C}_{{{{{{\rm{{Au}}}}}}}}^{l}\right)\\ 	=\frac{{\widetilde{D}}_{{{{{{{\rm{SiAu}}}}}}}}}{(1-{x}_{{{{{{\rm{{Si}}}}}}}})}\frac{1}{{V}_{m}^{l}{C}_{{{{{{\rm{{Si}}}}}}}}^{s}}\frac{\partial {x}_{{{{{{\rm{{Si}}}}}}}}}{\partial T}\nabla T$$

where *x*_Si_ is the mole fraction of silicon in the liquid alloy, *D*_Si_*, D*_Au_ are diffusion coefficients, ${\widetilde{D}}_{{{{{{\rm{{SiAu}}}}}}}}$ is the interdiffusion coefficient (*D*_Au_*x*_Si_ *+* *D*_Si_*x*_Au_), ${V}_{m}^{l}$ is the molar volume of the liquid alloy, ${C}_{{{{{{\rm{{Si}}}}}}}}^{s}$ is the molar concentration of pure solid silicon (superscript *l* is for liquid and corresponding parameters for gold have subscript Au), $\frac{\partial {x}_{{{{{{\rm{{Si}}}}}}}}}{\partial T}$ is the liquidus slope in the Si-Au phase diagram, and ∇*T* is the temperature gradient in the solid silicon core fibre across the droplet^44^. This model was used to derive droplet velocity predictions for several metals in Si^39^. Figure 2a shows calculated thermomigration velocities, normalised to the temperature gradient ∇*T* as function of inverse temperature for gold, gallium, and antimony^39^ through Si, as well as experimental values for two gold droplets from the present work, discussed in more detail below. While not all considerations (e.g. chemical potential) are included, the model provides guidance for design and interpretation of experiments. The relative values of the velocities for different metals should be valid in large core fibres as well as bulk solids, so for example, gold should be more rapidly transported through silicon than antimony given the same thermal gradient. The parameters indicate the relative ease of making structures using thermally driven transport in the fibre core. The thermomigration velocity measured for gold in the previous work, with a temperature gradient of 50 K cm^−1^, was 0.05 µm s^−1^ at a temperature of 930 °C. As the temperature and temperature gradient increase, improved solubility and transport of silicon across the gold alloy liquid region lead to more rapid translation of the liquid drop through the solid, with laser heated droplet velocities as high as 100 µm s^−1^. The temperature gradient in the laser treated material in this work is more than 100 times that in bulk studies at comparable temperatures^39^, yet the agreement between the model and these experiments is excellent, emphasising the applicability of a near-equilibrium thermodynamics description of the process.

**Fig. 2:**
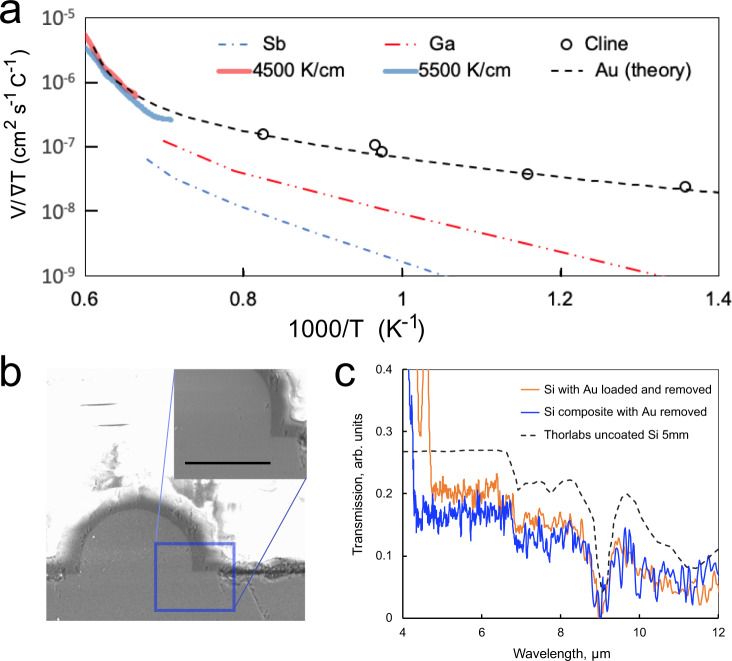
Thermomigration of Au in Si. **a** Calculated and experimental alloy droplet velocity in silicon normalised by the temperature gradient in the host, as a function of 1/*T* for several metals. Theory and data for Au in Si after Cline and Anthony^39,52^, with experimental data from two droplets in the present study (solid lines-orange for the gradient of 4500 K cm^−1^ and blue for the gradient of 5500 K cm^−1^). **b** Si fibre core welded to a single crystal Si substrate (using geometry of Fig. 1e) after thermomigration removal of a thin Au layer at the interface (inset close-up of weld region, scale bar 50 µm), and (**c**) Infrared transmission of Si fibres after removal of gold that was introduced by thermomigration (orange) and that was included in the preform when drawing the fibre (blue). The black dashed curve shows a scaled transmission profile of a 5 mm thick uncoated Si window (Thorlabs), to allow comparison of spectral features.

If sufficient solvent is accumulated in a confined geometry such as a fibre core, the melt will span the diameter of the core and the melt zone position and velocity can be controlled by the (laser) heat source. When the hot zone is translated slowly, the result is similar to travelling melt zone refinement. The travelling melt zone processing has been used in ingot refining (as the solubility of impurities is higher in the melt than the solid) since the 1950s^45^, for both elemental and compound samples and a more recent review^46^ emphasises its wide applicability. In semiconductor fibres, where unary fibres typically are drawn with polycrystalline cores, an alloying element can suppress nucleation^47^. This promotes the growth of long single crystals, even when compositional inhomogeneity is significant. During laser heating of these materials, thermomigration of liquid droplets can be observed^47^, and the accumulation of solvent to form a core-spanning melt zone can establish a TSFZ process, with the translation rate of the heat source chosen to optimise the formation of large grains. The choice of thermal gradient and annealing speed for fibre crystallisation, as noted by Badding^48^, is critical, and fundamental studies of thermomigration can provide important modelling information. With a melt zone travelling through an alloy, the scanning velocity relative to the thermomigration velocity will determine whether the droplets (and hence a compositionally enriched region) can match the speed of the heater, or if some material will lag, leaving a trail of solvent. The finite thermomigration velocity helps clarify the inhomogeneity of SiGe as-drawn material^47^; the thermal gradient of the drawing tower is small compared to that induced by the laser, and drawing speeds are higher- on the order of m min^−1^. SiGe forms a solid solution, so gradient composition structures are formed. For a eutectic system, any solvent residue will phase segregate upon solidification, with the cooling geometry and heat source velocity determining the arrangement of the segregated components. Si-GaSb and Si-Au as-drawn material have an inhomogeneous structure due to the rapid cooling of the draw.

In this work, a CO_2_ laser is employed to establish thermal gradients with varying geometrical profiles, and thermomigration and TSFZ processes were used to fabricate novel in-core structures in semiconductor core fibres. Thermomigration is employed to rearrange the constituents of the fibre core, and a travelling melt zone (which may or may not extend across the entire core) is used to recrystallise the material in a range of structures. For the first time to the authors’ knowledge, we demonstrate in-silicon formation of a III-V compound, and the use of laser-induced thermal gradients perpendicular to the axis of the fibre to create contiguous longitudinal electrodes and parallel semiconductor layers within the fibre core (Box 1).

**Fig. 3:**
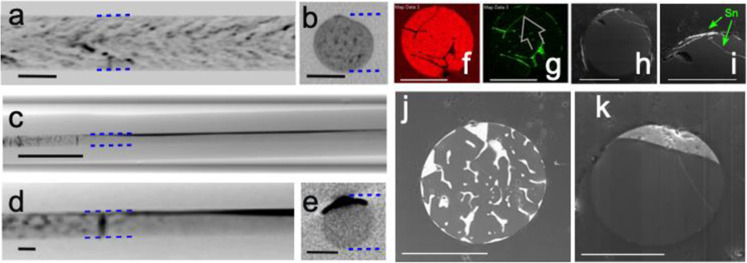
Lateral segregation of fibre components performed using the geometry of Fig. 1f. **a**–**e** are Si-Au, where dotted blue lines highlight the edges of the Si core and dark regions are Au; **f**–**i** are Si-Sn and **j**, **k** are Si-GaSb. **a** Side XCT of as-drawn Si-Au. **b** Cross sectional XCT of as-drawn Si-Au. **c** Overview XCT of Au-Si laterally segregated fibre; dark line is Au electrode. **d** Enlarged view of the transition between as-drawn and segregated core regions. **e** Cross-sectional XCT of the segregated region showing Au along one edge. **f** EDS map of Si in Si-Sn composite as-drawn fibre, **g** EDS of Sn in as-drawn fibre, with arrow showing the direction of Sn movement during segregation, **h** Overview BSE of Si-Sn core after segregation, showing the core has little residual Sn, and **i** closeup of Sn region showing extrusion into stress crack in the silicon, indicated by arrow. **j** BSE image of as-drawn Si-GaSb core fibre, and **k** Si-GaSb after segregation. Scale bars are 100 μm except in **c**, where the bar is 1 mm.

Box 1 Lateral segregation In eutectic multiphase fibres, whether as-drawn composites or pure fibres with a metal introduced by thermomigration, the gradients applied during subsequent processing determine the geometric distribution of the phases upon solidification. A strong transverse (perpendicular to the axis) gradient can be established if the fibre is thermally coupled to a substrate during laser treatment, as shown in Fig. 1f. With suitable translation speed of the heat source, thermomigration towards the laser and subsequent solidification can result in the formation of laterally segregated materials over centimetre distances. This segregation has been performed with as-drawn eutectic Si-Au, Si-Sn and Si-GaSb, as shown in the Fig. 3, below, as well as in fibres where the metal was thermally drawn into a pure silicon fibre, then segregated to one side locally.

**Au and Sn electrodes**

In the Fig. 3, panels a, b, show X-ray Computed Tomography (XCT) results for an as-drawn fibre with a 10 at.% Au in a Si core. Panel c presents an overview of the result of laser treatment with formation of a 4 mm long continuous filament of gold along the heated side. Panel d is a zoomed and contrast-enhanced view of the transition region between as-drawn and segregated material, and panel e is a cross sectional XCT of the core after treatment, showing the gold accumulated along one side. The transport of gold across the core is by a thermomigration mechanism, and the temperature gradient sufficient to cause transport across the fibre must be balanced by a laser translation rate that prevents the melt zone from spanning the entire core. It is worth noting that the thermal properties of the cladding-substrate system play a role in the ability to establish this balance, with heat capacity, coupling and cooling efficiency of the materials all of importance. For panels f–i, a 95 at.% Si, 5 at.% Sn fiber was attached to a graphite block, and heated on the other side to collect the Sn, as shown in panel i. For fibers with such low concentrations, a thicker electrode could be formed if metal was first agglomerated longitudinally during a slow scan with a melt zone that penetrated the entire core. An increase in speed then allowed formation of a metal electrode along one side of the core. Supplementary Information Fig. 4 shows a tin electrode formed using this method.

**Si-GaSb**

The lateral segregation method can also be used for fibres drawn with a mixture of semiconductors having a pseudo-eutectic phase diagram, such as Si-GaSb^18^. In Fig. 3j, k the formation of two distinct layers is demonstrated using this system. The power and scan rates were adjusted to allow thermomigration of the GaSb to the heated side of the fibre, and graphite was used to maintain the other side of the fibre below the melting point of pure silicon. Supplementary Movie 2 shows the segregation process. A thread of GaSb extruded into a stress-induced crack in the silicon core during solidification. This structure is a prototype for the formation of longitudinal diodes usable for solar conversion and photodetection. Preliminary investigation of longitudinal segregation for Bragg gratings are presented in Supplementary Information Fig. 5.

## Results and discussion

The velocity of Au droplets moving through Si core fibres was measured as a fundamental study to assure that at the large thermal gradients induced during laser treatment of silicon fibres, thermomigration is still adequately described by simple models. Metal addition, thermomigration and TSFZ were then used for recrystallisation and reconfiguration of the components of composite metal-semiconductor core fibres to demonstrate some possibilities for device fabrication.

### Metal introduction and transport: Au and Si

The use of an alloying element such as Ge in Si core fibre lowers the melting temperature of the core significantly and suppress nucleation during solidification, promoting the formation of longer single grain regions in the highly crystalline cores^47^. For materials with a low solid solubility in Si that form a eutectic (or pseudo-eutectic) rather than a solid solution, the added element (compound) can be removed during the recrystallisation process. Thermomigration removal of gold from a mixed Au-Si core was used in the fabrication of a high speed optical modulator for THz, but was limited to small sample length^49^. In the present work, production of longer samples was achieved by introduction of a small amount of gold into a silicon core fibre, translating to recrystallise and purify the material, then removing the metal. Controlling the locus of the alloy melt opens up new possibilities. Semiconductor photonics applications require waveguides typically made by etching a silicon-on-insulator structure, or increasing the concentration of Ge in a mixed-composition film epitaxially deposited on a silicon wafer^50^. Here, a metal film was used to weld an exposed fibre core to a Si substrate (with subsequent removal of the solvent phase), resulting in a rib waveguide of dimensions suitable for THz guiding. Metal introduction can also be used to modify the core either at doping or alloying concentrations. Supplementary Information Fig. 1 (GaAlSb) and 2 (Au_x_Ga_1−x_) document altered composition after thermal gradient introduction of metal elements to a semiconductor core fibre.

For fundamental studies of thermomigration, one end of a silicon core fibre, fabricated using the molten core method on a draw tower^51^, was coated with 500 nm of vapour-deposited Au (Fig. 1a). A slow increase of the laser power at a distance of 500 µm from the end of the fibre allowed microscopic observation of the dynamics of the thermomigration of small droplets of liquid through the core. Velocity measurements were extracted from the positional information in video frames acquired during the heating (see Supplementary Movie 1). As the silicon core is of high purity both before and after the transition of an alloy droplet, modelling the temperature and temperature gradient using the blackbody emission is a good approximation^27^, with the edge of the laser-induced melt zone of the pure core (ahead of the droplet) providing a temperature calibration point. The temperature gradient was found to be 4500–5500 K cm^−1^, and was approximately uniform over the observable travel range of more than 500 µm.

Velocity data normalised by the temperature gradient, from the trajectories of two particles, is shown in Fig. 2a as a function of temperature, demonstrating a high degree of repeatability. Also plotted are literature values of the gradient-normalised velocity at 50 K cm^−1^, and the theory from Anthony and Cline^44,52^. Their diffusion based model predicts the observed increase in slope at high temperatures, with good agreement between experiment and theory even at the temperatures and gradients imposed here. The laser heating results demonstrate the robust nature of Au alloy thermomigration, and the utility of simple models to predict the conditions necessary for processing.

Thermomigration with a solvent metal can be also used to form a bond between an exposed fibre core and a substrate, a method related to earlier wafer packaging work by Rudakov^37^. Here, the cladding glass was removed from one side of a piece of silicon core fibre containing 5% Sn (to reduce the temperature required for recrystallisation). This fibre was placed onto a gold-coated silicon substrate (Fig. 1e). With CO_2_ laser heating, the Au and Sn melted and were drawn away from the interface, leaving the Si fibre core welded to the substrate surface with no detectable interfacial discontinuity, as shown in Fig. 2b. In this manner, the use of a solvent layer may enable laser writing of out-of-plane waveguides^50,53,54^ with single crystal substrates as a crystalline template. Additionally, laterally segregated structures can be formed over centimeter distances, as described in Box 1.

Previous results on the gold-silicon system demonstrated enhanced infrared transparency of a silicon core fibre after a laser-based thermomigration gold extraction treatment^21^, which also recrystallised the Si. In that study, gold was removed from silicon (90 at.%)-gold (10 at.%) composite fibres, demonstrating the utility of a solvent for refinement of silicon core optical fibres. However, when the gold is a constituent of the drawn fibre, refined sample length is limited by the accumulation of gold at the laser focus; eventually the gold composition (for fixed laser power) increases until the Tiller criterion is violated^47^, and excess gold is deposited in the recrystallised Si. To overcome this limitation for the fabrication of long samples, a small bolus of gold was introduced into the core of a pure silicon fibre using the geometries of Fig. 1b, c. This allowed translation of the gold-rich melt zone along the host fibre for macroscopic distances, with a high degree of control over the translation speed of the solidification front and resultant crystal quality.

Figure 2c provides IR transmission results for a silicon core fibre recrystallised after thermomigration loading of a small amount of gold and subsequent translation of the laser-induced alloy melt zone a distance of 2.2 cm along the fibre. Also shown is data for a 1 cm composite Au-Si fibre from which gold was removed. The transparency of the bolus-refined sample suggests that this is a scalable method for production of far infrared transparent fibre. Supplementary Information Fig. 3 presents XRD results for the as-drawn and gold bolus-refined samples, showing the latter is composed of only two grains.

### Metal introduction and reaction: GaSb in Si

Using end-face or side-groove introduction, multiple elements can either be brought in sequentially from one side, or introduced from opposite directions as illustrated in Fig. 1d. This technique was used to synthesise inclusions of III-V compounds inside a silicon core. The introduction and translation of the constituent elements, which have eutectic temperatures in Si well below the melting point of the target compound, allows localisation of the reaction product.

**Fig. 4:**
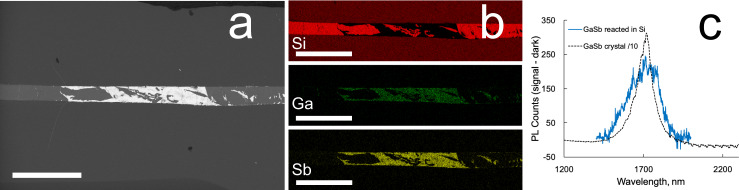
Results on GaSb reactively formed inside the Si core of a fibre at the midpoint of a 2 cm sample, using the geometry of Fig. 1d. **a** Backscattered SEM image of GaSb (bright areas) inside a silicon fibre core. Ga was thermally directed from the left and Sb was brought in from the right. **b** EDS results showing Si (top), Ga (middle), and Sb (bottom panel), demonstrating good segregation of GaSb from Si. Scale bars 500 µm. **c** GaSb photoluminescence measurements excited with 532 nm radiation for single crystal bulk material (dashed black curve; values divided by 10), and GaSb synthesised within the silicon fibre (blue curve).

Formation of a localised deposit of GaSb within a silicon core was demonstrated by first thermally drawing Sb through one end-face of a 2 cm long fibre, and subsequently drawing Ga into the other end. Each of the solvent metals was transported to the midpoint of the fibre using the laser to draw a full-width melt zone along the fibre to the reaction zone. A scanning electron microscope (SEM) image of the side polished fibre, along with energy dispersive (EDS) X-ray results and photoluminescence spectrum are presented in Fig. 4a, b. While the rapid cooling of the sample due to the pre-programmed laser power steps resulted in some commingling of the silicon and GaSb regions, the localised deposit suggests that a gentler cooling, accompanied by a slow translation, would result in silicon deposition followed by a block of GaSb. If an excess of one solvent metal is present, translation at lower power during cooling could be used to promote recrystallisation of the GaSb, in analogy with the Au crystallisation of Si. Residual metal could then be transported out through the side of the silicon through which it was introduced. In some samples, residual Sb (but not Ga) was observed by XCT (Supplementary Information Fig. 6) in the loading side of the fibre, likely associated with the slower diffusion of this material in Si (see Fig. 2a).

EDS results clearly demonstrate the presence of a mixture of GaSb and Si over a distance of 1 mm, and room temperature photoluminescence was observed from the reactively formed material, as shown in Fig. 4c. These results were obtained from the fibre that was polished for the SEM studies, to assure analysis of the same regions. Raman data (Supplementary Information Fig. 7) taken before polishing the fibre, confirm GaSb formation, and the lone transverse optical peak suggests a high degree of crystalline order.

This localised III-V deposit could be used as a biocompatible infrared source for spectroscopic applications, pumped with visible light through the cladding. An additional possibility raised by the assembly of the components from opposite ends of the fibre is that of creating a light emitting diode—with p-dopants and n-type dopants in the ends of the silicon and a compound light emitting material in the centre. A two-point measurement of the fibre resistance gave *R* = 10^5^ Ω, suggesting high doping levels. Production of this type of device will require more sophisticated control of the amounts of metal delivered to the reaction zone.

## Methods

The studies reported here were performed on silicon core, silica clad fibres with outer diameters from 0.5 mm to 1.5 mm and a core radius one tenth of the silica diameter. Fibres were made using the molten core draw method, with 3 cm silica preforms, and a temperature of 1950 °C. Pure silicon (*ρ* > 4800 Ω cm) core fibre was used for the metal introduction and recrystallisation studies, while 10 at.% Au, 5 at.% Sn in Si, and 30% GaSb in Si cores were used for lateral/transverse segregation of composites. Sn was introduced into a pure silicon core to demonstrate the feasibility of lateral segregation following metal introduction. Ga metal (99.995) and >99% purity Sn, Sb and Al foils from Alfa Aesar were used as metal sources.

A Bruker DaVinci powder diffractometer with a 15 mm diameter beam from a Mo K_*α*1,2_ (*λ* = 0.07093 nm, 0.071359 nm) was used in this work. The silica is sufficiently transparent at these wavelengths to permit analysis of the crystalline core without removal of the cladding. Samples were rotated at 1 Hz during Bragg scattering measurements, then strong reflections were analysed as a function of the fibre rotational position. XCT was performed using a Bruker Skyscan 1172 unit, at an acceleration voltage of 167 kV, source current of 59 µA, and image pixel size of 0.65 µm. IR data was obtained with a Bruker Tensor 27, using a PIKE Technologies beam condenser. Images and movies of thermomigration were made using a Thorlabs DCU224C CCD camera, and position vs. time information was extracted using Python software Trackpy^55^.

For metal introduction the metal of interest was either vacuum deposited (Au) or melted (Ga, Sn) onto the end of the silicon core fibre, or a thin foil (Au, Au + Sb, Sn, Sb, Al) was clamped between the fibre and a silica rod of similar diameter. The core of the fibre was then laser heated sufficiently to form a melt zone at a distance of 0.2 mm from the end of the fibre. For some metals (Ga, Au), thermomigration was efficient, with droplets of liquid travelling through the solid near the end of the fibre and a gradual increase in the size of the melt zone. For other elements, an oxide layer hindered metal uptake, and it was preferable to extend the melt zone to the end of the core, forming direct contact between the molten Si and the desired inclusion. Typical CO_2_ laser power levels were between 9 and 15 W, and scan rates were 10–20 µm s^−1^.

Travelling melt zone experiments via translation of the alloy melt zone were performed by relative motion of the fibre and laser beam, with typical velocities of 10–25 µm s^−1^, while maintaining a melt zone width of 0.5 to 2 mm. Slow reduction of power was used to minimise stress cracking as the core material solidified. Although the metal component might be expected to shrink and ameliorate the expansion of the silicon upon cooling, in eutectic systems the attractive interaction in the liquid phase leads to expansion of several percent upon solidification^56^.

Asymmetric heating was performed using a heat sink on the opposite side of the fibre from the laser irradiation. For radially symmetric laser illumination of novel core fibres, the source creates a melt zone that is uniform or nearly so across the diameter of the core. Even with unilateral heating, typical phase boundaries have an angle of greater than 75° with respect to the axis. To segregate the components transversally, one side of the fibre is thermally coupled to a graphite block while the laser is incident on the other. In this work, a 1 cm square cross-section graphite block was used to support the silica-clad fibre. To assure uniform thermal contact, a v-groove of depth 0.5 mm was machined along the block. This groove was loaded with a small amount of Aquadag^®^ graphite liquid suspension into which the fibre was placed. The power level, spot size and translation rate were adjusted to allow thermomigration from the cold side of the fibre, without forming a melt zone that extended across the full core diameter. Laser spot sizes up to 1 mm were used in combination with translation rates up to 50 µm cm^−1^ for fibres of 0.5–1.3 mm outer diameter.

## Supplementary information

Supplementary Information

Supplementary video 1- Gold alloy thermomigration in silicon

Supplementary video 2- Thermomigration segregation of Si/GaSb core fibre

Inventory of Supporting Information

## Data Availability

The dataset for Fig. 2 can be found on Zenodo (10.5281/zenodo.6095549).
