# Supplementary material for: Localised structuring of metal-semiconductor cores in silica clad fibres using laser-driven thermal gradients
Source: Nat Commun. 2022 May 13;13:2680. doi: 10.1038/s41467-022-29975-1 (PMC9106754; doi:10.1038/s41467-022-29975-1)
Supplement: Supplementary file 1 — Supplementary Information [file 41467_2022_29975_MOESM1_ESM.docx]

Supplementary material

**Contents:**

**Al doping of GaSb 1**

**Reactive formation of Au-Ga alloys 2**

**XRD of Au recrystallized Si 3**

**2-step formation of Sn electrode in Si 4**

**Periodic structuring of eutectic alloy fibre cores 5**

**GaSb reactively formed in Si 6**

**References 7**

**Al doping of GaSb**

A piece of Al foil was clamped against the end of a hand-drawn GaSb core fibre, and the end of the fibre was laser heated until core melting was observed. The laser was then translated over 1 mm. The end of the fibre was polished and EDX analysis was performed, with the results shown In Fig 1. Compositional results demonstrated the inclusion of sufficient Al that a bandgap increase would be expected. In these preliminary studies, up to 20 at% Al inclusion was observed, with a reduction in the Ga content corresponding to the increase in Al.

**
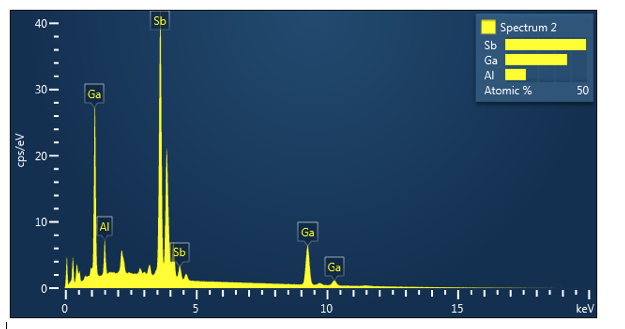
**

Element Atomic %

Al 12.67

Ga 37.87

Sb 49.46

Total: 100.00

Figure 1 EDX results on the material formed by introduction of Al into a GaSb fibre, with compositional information

**Reactive formation of Au-Ga alloys**

Gold-gallium alloys are employed as microelectronic interconnects, due to the low melting temperatures for bonding^2^ with, e.g. Au_2_Ga having a melting point of ~350C ^3^, combined with robust mechanical properties. Ga-Au compounds were reactively formed within the silicon core, introducing first Ga (eutectic point ~29.7 C) and then a gold 99\%- antimony1\% alloy (Sigma Aldrich) through the same end of the fibre. Ga-Au intermetallics with varying composition and morphology were formed. SEM/EDX results in Fig 2 show a mixed phase region with Si and Au-Ga compounds (with a high concentration of Au). Sb assisted in wetting of the silicon.

Figure 2 Alloy formation within a Si host fibre. a) SEM image of the eutectic structure formed by the combination of Si, Au and Ga (Trace amounts of Sb). b) EDS results suggest the formation of a mixture of Au and βAu_2_Ga_2_


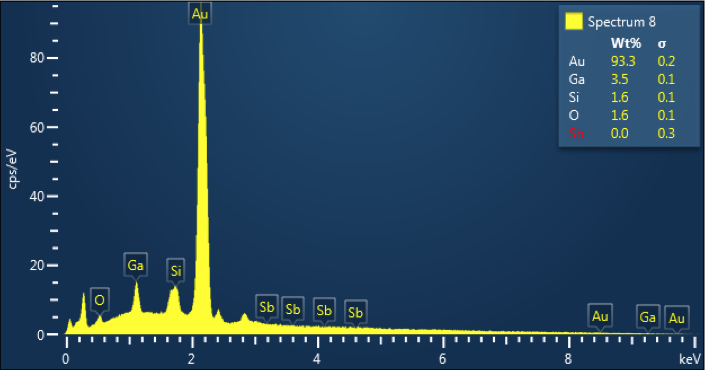

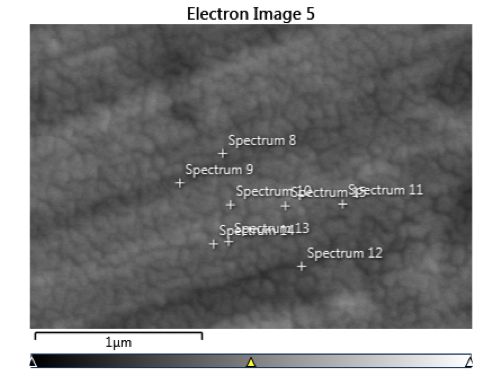


a b

**XRD of Au recrystallized Si**

Figure 3 shows the results of XRD scans, as described in Song, et al^1^. Bragg peaks indicate only Si, with a smaller number of peaks after recrystallization. Phi scans indicate the presence of only two grains over the scanned length (2.2 cm) of the sample


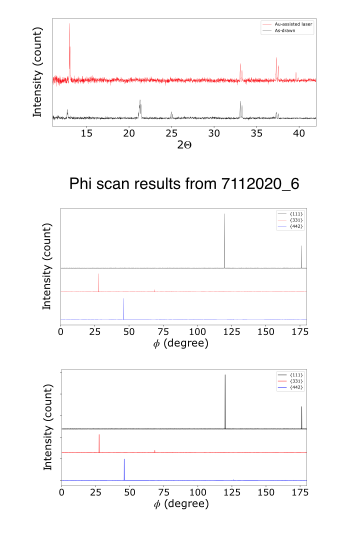


a

b

Figure 3 Xray diffraction results on pure silicon fibre and a fibre into which Au was introduced, scanned, and removed. a) Bragg results showing fewer peaks for Au-refined material b) phi scan results from the refined fibre showing small number of reflections associated with two crystalline grains.

**2-step formation of Sn electrode in Si**

To form Sn electrodes of greater thickness, tin was aggregated by scanning a 5% Sn fiber at 19W, 20 μm s^-1^ for a distance of 12.8 mm. The resulting plug of Sn was then used as a source to make a thick lateral electrode over 2.2 mm, using a scanning speed of 50 μm s^-1^.

**Fig. 4** XCT images of thick Sn electrode formed on the side of a 5 at% Sn, 95at% Si fiber. Scale bar 1mm

**Periodic structuring of eutectic alloy fibre cores**

Previous studies on SiGe fibers demonstrated the possibility of laser inscription of grating structures^4^ in SiGe (solid solution) core fibres. To assess this possibility in eutectic systems, a section of GaSb-Si composite fibre was scanned using a series of 1s pauses every 500 microns, during a 1mm s^-1^ scan at 24 W power of the engraver laser. XCT results from this propaedeutic experiment demonstrate the possibility of making periodic structures in (pseudo) eutectic systems. The scan resulted in uneven accumulation of the material; GaSb was cleared from ~100 microns on the side ahead of the translation pause, but incomplete recruitment over the 400 microns between steps is evident. The incomplete thermomigration of the rapidly scanned regions suggests that the scan rate was too high, or the dwell time was too short. Scanning backwards a short distance, or placing the pause sections at 100 micron separation (for the fibre and power level used) would have improved the removal of GaSb from the silicon. Use of a smaller core fibre will allow writing of shorter period Bragg gratings, due to more rapid cooling. Scale bar 500 μm. Contrast of original x-ray image increased to improve clarity.


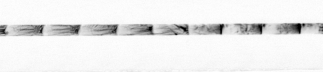


Fig. 5 XCT image of first attempt at formation of long period grating in a Si-GaSb pseudo eutectic fibre

**GaSb reactively formed in Si**

Additional information on the GaSb formed by sequential addition of the elements to a silicon core fiber is presented here. XCT (Fig 5) shows clean silicon in the wake of the Ga translation, but residual metal in the position to which Sb was drawn in the first introduction step. Raman results (Figure 6) show only one peak for each constituent, suggesting a high degree of crystalline orientation^5^.


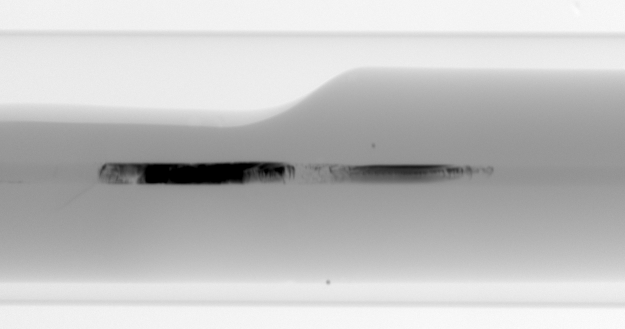


GaSb Sb residue

**Figure 5** XCT image of reacted GaSb in the silicon fibre. Sb was first loaded from the right, then Ga was brought in from the left. Scale bar 1mm. Contrast of original x-ray image increased to improve clarity.


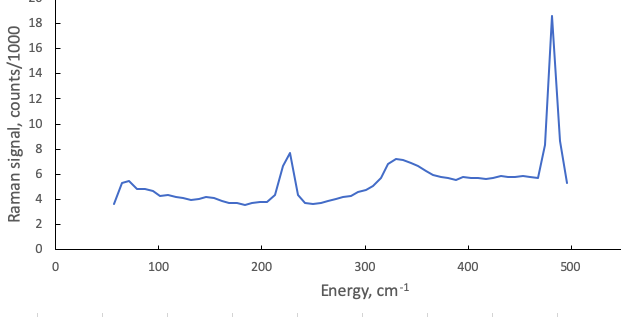


**Figure 6**  Raman spectra taken on the region of the fibre into which Ga and Sb were drawn clearly shows the peaks for silicon (shifted from the position for pure material, as well as the GaSb TO peak^5^ at ~229 cm ^-1^

References

1. Song, S. *et al.* Laser restructuring and photoluminescence of glass-clad GaSb/Si-core optical fibres. *Nature Communications* **10**, 1790 (2019).

2. Frömel, J., Lin, Y.-C., Wiemer, M., Gessner, T. & Esashi, M. Low temperature metal interdiffusion bonding for micro devices. in *2012 3rd IEEE International Workshop on Low Temperature Bonding for 3D Integration* 163–163 (2012). doi:10.1109/LTB-3D.2012.6238080.

3. Okamoto, H. Au-Ga (Gold-Gallium). *J. Phase Equilib. Diffus.* **34**, 174–175 (2013).

4. Coucheron, D. A. *et al.* Laser recrystallization and inscription of compositional microstructures in crystalline SiGe-core fibres. *Nature Communications* **7**, 13265 (2016).

5. Svendsen, S. Compositional characterisation of optical InGaSb-core microfibres. (NTNU, 2017).
