# Supplementary material for: Localised structuring of metal-semiconductor cores in silica clad fibres using laser-driven thermal gradients
Source: Nat Commun. 2022 May 13;13:2680. doi: 10.1038/s41467-022-29975-1 (PMC9106754; doi:10.1038/s41467-022-29975-1)
Supplement: Supplementary file 4 — Inventory of Supporting Information [file 41467_2022_29975_MOESM4_ESM.docx]

Inventory of supporting information

Supplementary information file (pdf)

Supplementary movie 1

Supplementary movie 2

descriptions

Supplementary movie 1: Gold-silicon alloy droplet moving through silicon core fibre towards the high temperature region.

Supplementary move 2: GaSb rich droplets thermomigrating to the top (heated) side of a silicon core fibre. This results in segregation of the two materials along the length of the treated length.
